# Establishing a Community Council to Enhance Arab American Engagement in Dementia Research

**DOI:** 10.1093/geroni/igaf122.144

**Published:** 2025-12-31

**Authors:** Sara Masoud, Howaida Werfelli, Caitlin Sangdahl, Azizeh Sowan, Amani Charaf, Fayron Epps

**Affiliations:** UT Health San Antonio, San Antonio, Texas, United States; Salvere Public Health Consultants, San Antonio, Texas, United States; UT Health San Antonio, San Antonio, Texas, United States; UT Health San Antonio, San Antonio, Texas, United States; UT Health San Antonio, San Antonio, Texas, United States; UT Health San Antonio, San Antonio, Texas, United States

## Abstract

Arab Americans face a disproportionately high risk for Alzheimer’s disease and related dementias (ADRD) but remain severely underrepresented in dementia research. Systemic barriers—including race/ethnicity misclassification, institutional exclusion, and structural discrimination—contribute to this underrepresentation, limiting the development of culturally responsive interventions. An engaged approach is necessary to ensure that research aligns with the experiences and priorities of communities most impacted by dementia. Anchored in emergent engagement science frameworks and the principles of Community Based Participatory Research (CBPR), this presentation outlines steps and strategies used to establish and build capacity for co-conducting research among community members and researchers. A Community Council (CC) (N = 9) for Arab Dementia research was established. Strategies for establishing the CC included: 1) organizing a community convening to initiate action around ADRD research disparities; 2) monthly meetings and working sessions; and 3) capacity building activities (e.g., discussing basics of research, co-planning convenings outside of the research study deliverables) to strengthen the potential for a successful research partnership. Within each strategy, approaches to overcome challenges (e.g., recruiting male CC members, balancing competing priorities) encountered will be discussed. Research conducted through this community-academic partnership will contribute to building evidence around innovative engagement science frameworks, raising awareness about the research needs and experiences of the Arab American community, and toward developing and testing models of engagement scalable to other populations.

